# Multimode fibre based imaging for optically cleared samples

**DOI:** 10.1364/BOE.8.005179

**Published:** 2017-10-23

**Authors:** Ivan Gusachenko, Jonathan Nylk, Javier A. Tello, Kishan Dholakia

**Affiliations:** 1 SUPA, School of Physics and Astronomy, University of St. Andrews, Fife, KY16 9SS, UK; 2 School of Medicine, University of St. Andrews, Fife, KY16 9TF, UK

**Keywords:** (070.6120) Spatial light modulators, (110.2350) Fiber optics imaging, (110.7348) Wavefront encoding, (170.3660) Light propagation in tissues, (180.5655) Raman microscopy, (180.2520) Fluorescence microscopy

## Abstract

Optical clearing is emerging as a popular approach particularly for studies in neuroscience. However the use of corrosive clearing solutions typically requires sophisticated objectives or extreme care with optical components chosen for single- or multi-photon imaging. In contrast to the use of complex, custom-made microscope objectives, we show that the use of a corrected multimode fibre (MMF) offers a route that is resistant to corrosion, can be used in clearing media, is not constrained by the refractive index of the immersion medium and offers flexible working distances. Using a corrected MMF, we demonstrate fluorescence imaging of beads and stained neuroblastoma cells through optically cleared mouse brain tissue, as well as imaging in an extreme oxidative environment to show the versatility of our approach. Additionally, we perform Raman imaging of polystyrene beads in clearing media to demonstrate that this approach may be used for vibrational spectroscopy of optically cleared samples.

## 1. Introduction

Volumetric imaging methods are enabling researchers across the biomedical sciences, particularly in neuroscience, to visualize large tissue sections and sample volumes in three dimensions. A key development in the preparation of biological tissues for such imaging is optical clearing, a chemical treatment for homogenization of specimen refractive index (RI). This facilitates the visualization of macroscopic tissue sections, whole organs, and even of entire animals [1–6].

Optical clearing has the potential to revolutionize large scale structural microscopy but a number of challenges must be solved before the benefits can be fully appreciated. Perhaps the most significant and overlooked problem with optical clearing is the suitability of optical components (e.g. the microscope objective lens) for immersion in the clearing solution. Organic solvents, which yield the best sample transparency [2], are the most aggressive and can cause damage to the objective lens. In particular, to the best of our knowledge there is no suitable objective lens for immersion in benzyl alcohol and benzyl benzoate (BABB) solution [7] or dibenzyl ether (DBE) solution [8,9] with a working distance suitable for large volume imaging [2].

An approach to circumvent this problem is to use an objective designed for operation in air and perform observations external to the sample chamber. However, this introduces strong spherical aberration, greatly reducing the image quality that can be achieved, which can be partially mitigated by the introduction of specialist optical components into air objectives [4]. While there is pronounced commercial development in clearing-compatible objective lenses, the cost for such lenses is high and cannot be expected to drop below the cost of a conventional immersion lens. Additionally, it is not possible that a single objective lens will be fully compatible with all clearing techniques (different clearing methods work better for different imaging methods [2]) and offer the flexibility needed for all operating parameters during imaging (e.g. numerical aperture (NA), field-of-view (FOV), working distance).

A more powerful approach would be to exploit a complex medium of monolithic design; a representative example of such a medium is a bare multimode fibre (MMF). Such fibers, made mostly of silica or inert polymer, offer corrosion resistance and are compatible with a wide range of immersion media. Additionally, they allow for aberration-free imaging in media with different RI. However the fibres are not specifically designed for imaging applications due to modal interference resulting in the scrambling of the propagated light wavefront, that yields a speckle pattern. However, recent advances in wavefront shaping [10–12] with dynamic diffractive optical elements (e.g. spatial light modulators (SLMs)) have enabled multimode fibres to be used as imaging objective lenses and novel endoscopes. This has included many different microscopy modalities such as reflection [13], dark field [14], fluorescence [14], two-photon excited fluorescence [15, 16] and Raman scattering [17]. There is also the prospect of using MMFs for light-sheet imaging [18]. Importantly, such results are achieved in the absence of any additional optics at the fibre output. This approach can be regarded as deferring the image formation function to the wavefront shaping system, relaxing the stringent conditions on the design of immersion objectives. Rather than addressing endoscopic applications, the focus of this paper is to address a major unmet biophotonics need for *ex vivo* studies.

The originality of this paper is to use the fibre as an immersion objective which is structurally and chemically robust, and may be used with any refractive index value for the immersion medium. Specifically, we demonstrate the use of MMFs for imaging in two optical clearing solutions: 2,2′-thiodiethanol (TDE) [1,3] and Visikol-HISTO-1. We experimentally show that the optical resolution remains unchanged as a function of the clearing medium’s RI. With our fibre microscope we obtain fluorescence images of neuroblastoma cells placed behind a section of optically cleared mouse brain tissue. Furthermore, we show the versatility of our approach for a range of diverse applications and environments by demonstrating fluorescence imaging in a highly corrosive oxidative environment (concentrated sulfuric acid), and finally demonstrate Raman imaging of polystyrene beads in TDE.

## 2. Materials and methods

### 2.1. Experimental setup

**Fig. 1:**
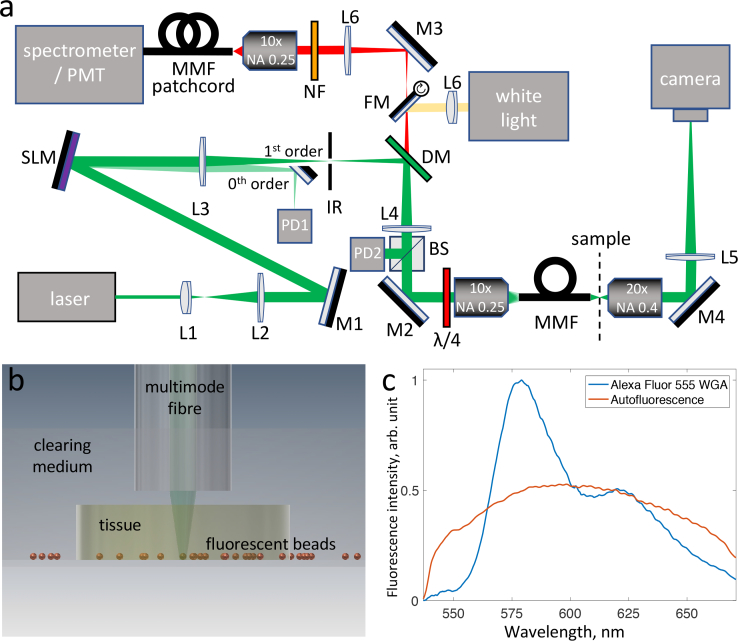
(a) Schematic of the experimental setup. Lx - lenses, Mx - mirrors, IR - iris, DM - dichroic mirror, FM - flip mirror, NF - notch filter, BS - 50:50 beamsplitter, PDx - photodiodes. (b) Schematic of the sample chamber, showing fluorescent beads covered with a TDE-cleared brain tissue and immersed in TDE. (c) Representative emission spectra of Alexa Fluor 555 and the tissue autofluorescence, when excited at 532 nm. The plots are normalized to have identical areas under each curve.

The experimental setup shown in Fig. 1(a)

is almost identical to our previous work and is described in detail elsewhere [17]. The wavefront of a CW laser beam with *λ* = 532 nm (Verdi-5V, Coherent, Santa Clara, CA) was shaped by a spatial light modulator (LCOS-SLM X14168-01, Hamamatsu Photonics, Hamamatsu, JP) and fed into a multimode fibre (Thorlabs, AFS50/125Y, low-OH, 50 μm core, NA 0.22, 40 mm length), which was placed conjugate to the Fourier plane of the SLM. The light from the fibre output illuminated the sample, and the emitted fluorescence (or Raman) signal was collected using the same MMF probe used for illumination. A set of 532 nm Raman dichroic mirror/notch filters (DM, RasorEdge 532 dichroic and NF, 532 notch, Semrock, Rochester, NY) was placed into the detection beam line before coupling the light into a MMF patch cord, either fed into a spectrometer (Shamrock 303i with Newton 920, Andor, Belfast, UK) or a photomultiplier tube module (H6780-20, Hamamatsu, Hamamatsu, Japan). The photodiode PD1 provided synchronisation for the fluorescence microscopy, and PD2 monitored the power at the distal end of the fibre.

Wavefront correction through a MMF is an established experimental technique, which relies on prior calibration of the system. The speckle patterns emanating from the fibre were recorded in a transmission geometry using a CCD camera (piA640-210gm, Basler, Ahrensburg, Germany) and were used to measure the fiber transmission matrix (TM). The specific details of the correction procedure implementation, as well as discussions on the attainable FOVs and correction efficiencies are given in our previous work [17]. The phase masks obtained as a result of the calibration were displayed onto the SLM to focus the light into diffraction-limited spots either 50 μm or 450 μm behind the proximal facet of the MMF, resulting in a field of view (FOV) of 50 μm and 200 μm, respectively. The fibre TM is sensitive to the exact fibre geometry, but the bending sensitivity is not an issue for our application *ex vivo*. For endoscopic use this issue may be addressed by rapid recalibration of the system using digital mirror devices [19], or potentially by numerical calculation of the TM based on known fibre deformation [20].

For the spectral acquisitions, we used a blazed grating operating at 500 nm (600 lines/mm), and the entrance slit of the spectrometer was set to 200 μm (measured FWHM spectral resolution was 32 cm^−1^ at 1000 cm^−1^ and 25 cm^−1^ at 3000 cm^−1^).

For imaging samples underneath cleared tissue, the samples were placed into a glass bottom (150 μm thickness) petri dish, then a slice of cleared tissue was placed on top. Finally the sample and tissue was immersed in clearing medium (see Fig. 1(b)).

### 2.2. Effect of clearing/immersion media on the fibre

**Fig. 2:**
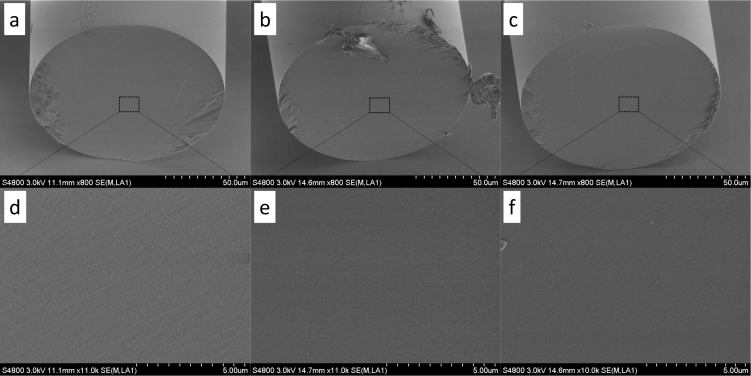
SEM images of freshly cleaved fibre (a, d), and fibres after 24h immersion in TDE (b, e) and sulfuric acid (c, f). (a–c) show the entire facet, while (d–e) are close-up images of the central part of the core.

To assess the effect of TDE and sulfuric acid on the fibre, we immersed cleaved fibres into both these media for 24 hours. The fibres were then washed with ethanol, dried, and imaged with a scanning electron microscope (Hitachi S-4800 SEM, Tokyo, Japan). The images are shown in Fig. 2

. The results show the exposure to the chemicals did not have any detrimental effect on the fibre (see Figs. 2(e, f)), apart from smoothing sub-micrometer scale surface roughness, which is seen on the untreated cleave (see Fig. 2(d)). Overall, immersion for up to 24 hours did not affect the ability to perform focus correction and imaging through the fibre.

### 2.3. Optical resolution with clearing media of different refractive index

Solutions were prepared by mixing TDE (RI*_TDE_* = 1.52) with phosphate buffer saline (PBS, RI*_PBS_*=1.34) in different proportions, to obtain solutions with a range of RIs. Additionally, the resolution was measured for Visikol HISTO-1 clearing medium with RI_*HISTO*−1_ = 1.50. After immersion, the calibration procedure was performed 30 μm away from the fiber facet. Then, images of two different spots near the centre of the fibre was acquired with the camera and fitted with an Airy disk function to obtain the NA. More precisely, the image of the spot was fitted with the function (2*J*_1_(*r/a*)/(*r/a*))^2^ to extract the scaling parameter *a*. Here, *J*_1_(·) denotes the first order Bessel function of the first kind, and *r* is the radial coordinate. The NA is related to *a* by NA = *λ*/(2*πa*). The errors were calculated based on two different focused spots near the centre of the FOV.

### 2.4. Fluorescence microscopy

Fluorescence imaging was performed by digitally scanning a focal spot at the output of the fiber. Intrinsically low dark noise of the electron-multiplier detector is highly beneficial and therefore it is preferred for many fluorescent imaging applications; in this work it was used for imaging of fluorescent beads. It might be difficult however to reconstruct images of relatively weakly fluorescent stained cells in the presence of strong autofluorescence. On the other hand, the careful choice of the fluorescent dye allows us to circumvent this problem by spectrally differentiating the dye emission from the autofluorescence. Namely, Alexa Fluor 555 used here, has a different emission spectrum compared to the autofluorescence when excited at 532 nm, and notably has very low emission below 550 nm (see Fig. 1(c)). Therefore, we used spectral detection to image labelled cells. Details of PMT- and spectrometer-based acquisition processes are given in Appendix A2. The power at focus was estimated to be 15 μW when imaging 50 μm from the fibre (Fluorescence imaging in a hazardous environment), and 500 μW when imaging 450 μm from the fibre (Fluorescence imaging through cleared tissue).

### 2.5. Raman imaging

Raman imaging was performed by digitally scanning a focal spot at the output of the fiber, with Raman signal collected back through the MMF into the spectrometer, as described previously [17]. The Raman spectra were acquired with a 3s dwell time per pixel. The power in the corrected focus was ∼40 mW. The acquired Raman spectral images were then processed as described in Appendix A1.

### 2.6. Sample preparation

#### 2.6.1. Optically cleared brain tissue slices

*Animal care*. All rodent experiments were approved by The Home Office and the University of St Andrews, under the consultation of a veterinary surgeon, using Dr J Tello’s Project Licence 70/7924 in accordance with the ASPA guidelines. Mice were bred and housed at the University of St Andrews, St. Mary’s Animal Unit under regular light-dark cycles (12h light, 12h dark) under constant temperature 22 ± 2 °C with food and water available *ad libitum*. The genetic mouse line used in these experiments were *Kiss1*^tm1.1(cre/EGFP)Stei^/J mice [21].

*Tissue preparation*. Adult heterozygous mice were anaesthetised with an overdose of sodium pentobarbital (100 mg/kg), then transcardially perfused with 0.1M PBS (pH 7.4) followed by 4% paraformaldehyde (PFA) in PBS (pH 7.4). The skull was opened and the brains were removed and post-fixed overnight in 4% PFA in PBS at 4 °C. The brains were cryopreserved in 30% sucrose in 0.1M PBS. Brain sections (300 μm thickness) were obtained using a Compresstome vibratome (Precisionary Instruments, VF-300). Tissue sections were optically cleared using TDE as described by Costantini et al [1]. The clearing solution was also used as the immersion medium for the imaging, which is consistent with other protocols for imaging in cleared samples.

#### 2.6.2. Fluorescently-labeled neuroblastoma cells

Wild-type SH-SY5Y (neuroblastoma) cells were cultured in Dulbecco’s Modified Eagle Medium: Nutrient Mixture F-12 (Gibco), supplemented with 10% fetal bovine serum and 1% Penicillin/Streptomycin, and grown in a humidified atmosphere of 5% CO_2_, at 37°C. They were seeded into 35mm glass bottom dishes using TrypLE express enzyme solution (Gibco) at a density of 10^6^ cells per dish in 3mL of complete media. After 24 hours, the cells were washed twice with PBS and fixed using 4% PFA for 15 minutes at room temperature (RT), after which they were washed three time with PBS and labeled using a Wheat Germ Agglutinin conjugate of Alexa Fluor^TM^ 555 (Thermo Fisher Scientific). The label was applied to cells at a concentration of 5 μg/mL in PBS and incubated for 10 minutes at RT. Cells were then washed three times in PBS and stored in PBS at 4°C until needed for imaging.

Immediately before imaging, the cells were drained, then a slice of a cleared brain tissue placed on top of the cell layer. Residual TDE was removed with a lens cleaning tissue, then the slice was attached punctually on its perimeter with a few drops of cyanoacrylate (“superglue”) and left to dry for 10 minutes. After this, the dish was filled with 100% TDE to cover the tissue, and agitated manually for 5 minutes to remove any RI gradients, as assessed by a naked eye.

#### 2.6.3. Microspheres

Suspensions of appropriate microspheres (beads) were added to 100% ethanol (1% v/v), deposited in a glass bottom (150 μm thickness) petri dish or on a #1 glass coverslip, and dried with compressed air. We used 2 μm diameter red fluorescent polystyrene beads (Duke Scientific Corp. R0200) for fluorescence imaging and 11 μm diameter polystyrene beads (Thermo Scientific 7510A, ≤ 18% coefficient of variation) for Raman imaging. The dried beads were immersed in an appropriate immersion medium (TDE or concentrated sulfuric acid (95%, Fisher Scientific S/9240/PB17)) and were imaged typically within two hours.

## 3. Results

### 3.1. Optical resolution with clearing media of different refractive index

The optical resolution of the fibre microscope was measured in clearing solutions with varying RI at a distance of 30 μm from the fibre facet. The measured resolution and NA is summarized in Table 1

**Table 1:** Numerical aperture and resolution for different refractive index compounds, for *λ* = 532 nm.

Clearing solution	RI	Resolution (0.61*λ*/NA), μm	NA

PBS	1.34	1.59±0.04	0.205±0.006
25% TDE	1.38	1.52±0.06	0.214±0.009
50% TDE	1.43	1.51±0.03	0.215±0.004
75% TDE	1.47	1.53±0.03	0.212±0.004
100% TDE	1.52	1.53±0.03	0.212±0.005
Visikol HISTO-1	1.50	1.50±0.03	0.216±0.005

. A diffraction limited spot could be achieved independently of the medium RI, with the resolution only dictated by the fiber NA. This behaviour is expected, as the NA is imposed by that of the fibre, which is fixed. Higher RI would allow for a tighter focus for a given angle of beam convergence (NA = *n* sin (*θ*)). However, in the case of the immersed fiber, the associated increase of the RI is exactly compensated by a decrease of the angle of convergence, as follows from Snell’s law.

### 3.2. Fluorescence imaging through cleared tissue

**Fig. 3:**
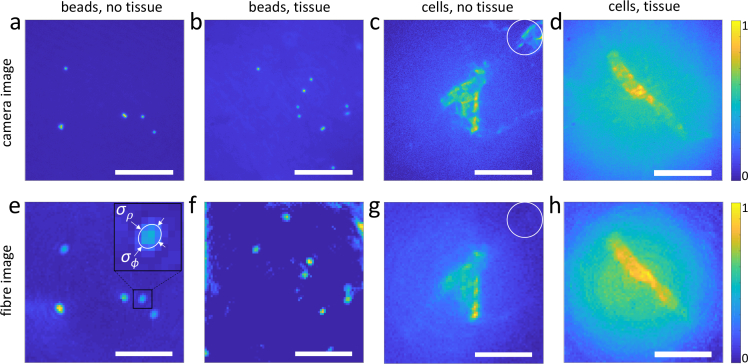
Fluorescence images of fluorescent beads (a, b, e, f) and labelled neuroblastoma cells (c, d, g, h). Reference images recorded with the camera (a–d) and through the MMF (e–h). The samples were imaged directly (a, e, c, g) and through a slice of a tissue cleared with 100% TDE (b,d,f,h). The inset in (e) shows a zoomed-in image of a single bead, used to determine its radial (*σ_ρ_* ≈ 4.2 μm) and azimuthal (*σ_ϕ_* ≈ 4.9 μm) FWHM width. Scale bars are 50 μm.

Fluorescence images of beads and labelled neuroblastoma cells are shown in Fig. 3

. The transmission matrix was measured in a region of the petri dish away from any beads, cells, and tissue, and then images were recorded either of unobstructed sample (see Fig. 3(a,c,e,g)), or sample covered with a cleared brain slice (see Fig. 3(b,d,f,h)). The sample was placed about 450 μm from the fibre, which resulted in measured resolution of ≈ 2.8 μm (FWHM, data not shown), in accordance with our previous results [17]. The width of a 2 μm bead image shown on the inset of Fig. 3(e) (FWHMs *σ_ρ_* ≈ 4.2 μm radial and *σ_ϕ_* ≈ 4.9 μm azimuthal) is consistent with the expected value of 3.8 μm (FWHM of an Airy disc convolution with a 2 μm-wide rectangle function). We attribute the ellipticity and the slightly coarser resolution of the PSF to partial mode excitation, as described in [22]. It can be seen that images of beads behind a tissue slice show higher background due to the strong autofluorescence from the tissue (see Fig. 3(b,d,f,h)). For the samples underneath tissue, we also observed an increased background signal in the fibre obtained images (see Fig. 3(f,h)) compared to the reference camera images (see Fig. 3(b,d)) as a consequence of collecting the fluorescence back through the tissue when imaging with the fibre. The cell feature (white circles on Fig. 3(c,g)) is seen on the camera, but not on its fibre image counterpart, as this feature is effectively outside the FOV of the fibre microscope due to focusing and collection efficiency degrading with radial distance.

### 3.3. Fluorescence imaging in a hazardous environment

To demonstrate the capability of our system to withstand extreme conditions, we performed fluorescence imaging of 2 μm diameter fluorescent polystyrene beads in concentrated sulfuric acid (H_2_SO_4_). This is a strong oxidiser, and is corrosive towards aluminium alloys, titanium, copper and some stainless steels. However, both polystyrene and silica glass are resistive against sulfuric acid. A potential original application for such imaging in an oxidative environment would be to visualise acidophilic microorganisms, with a notable example of *H. Pylori*—bacteria causing stomach ulcers and increasing risk of gastric cancer [23].

**Fig. 4:**
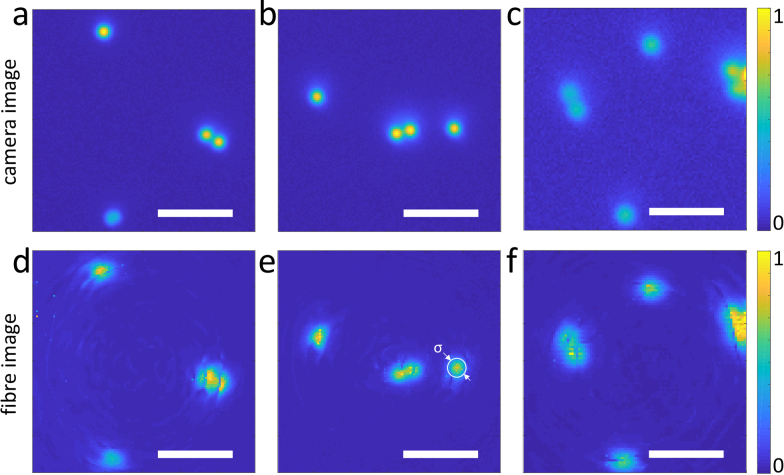
Fluorescence images of 2 μm fluorescent beads immersed in concentrated sulfuric acid. (a–c) camera (reference) images, (d–f) MMF images. The white circle on (e) shows the FWHM *σ* ≈ 2.0 μm of a bead image intensity. Scale bars are 10 μm.

The images are shown on Fig. 4

. Beads imaged through the fibre were measured to have a FWHM of 2.0 μm, in good agreement with the expected resolution of the fibre microscope. The dark rings in some of the fibre images are a consequence of using an internal reference for determination of the transmission matrix [10]. These results confirm that MMFs are suitable for imaging media where standard objectives would not work.

### 3.4. Raman imaging in clearing medium

Figure 5

**Fig. 5:**
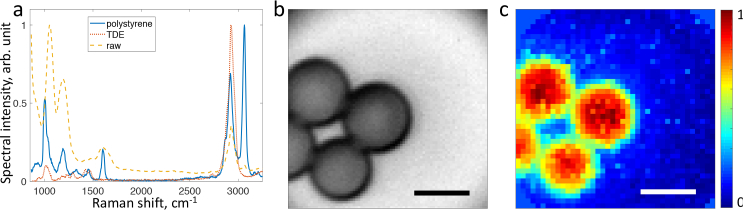
Raman imaging of polystyrene beads in TDE. (a) A typical raw Raman spectrum dominated by the fibre background (yellow dashed), and extracted Raman spectra of polystyrene beads (blue solid) and TDE immersion (red dotted). (b) Bright field (reference) image of polystyrene beads. (c) Raman image of polystyrene beads, as shown by the intensity of the correspondent spectral component from (a). Scale bars are 10 μm.

shows Raman spectra and images of polystyrene beads immersed in TDE. The Raman spectrum of polystyrene can be identified despite the strong contamination of Raman background of the MMF [17] and the immersion medium (see Fig. 5(a)). High background autofluorescence and weak intrinsic tissue Raman signal currently prevents tissue Raman imaging with our system. To circumvent this in future, prolonged tissue bleaching prior to imaging [24] may be considered.

## 4. Discussion

We have shown that a MMF can be effectively used as an objective for imaging in optical clearing solution. In contrast to microscope objectives, which are usually designed to work at a particular RI of the immersion medium, the fibre is insensitive to changes in RI. We have successfully demonstrated cellular fluorescent imaging with cleared tissue, Raman imaging in TDE, and fluorescent imaging in concentrated sulfuric acid, with no damage or adverse effects suffered by the fibre.

The resolution of the current system is relatively low, and is limited by the NA of the MMF and the distance between the fiber and image plane: the effective NA is 0.22 in the proximity of the fibre, and ∼0.075 at 450 μm from the fibre when immersed in 1.5 RI solution. The resolution is thus tunable as the fiber-image plane spacing can be adjusted. Importantly, we note that high-NA fibers are now becoming more widely available [25].

As can be deduced from previous work [14,17], the achievable FOV, defined as the diameter of the largest region with a uniform effective numerical aperture NA*_eff_* at a distance WD , is given by:

(1) $$\emptyset \text{FOV}=2\left|\text{WD}/\text{tan}\;\theta -R\right|,\;\text{with}\;{\text{NA}}_{eff}=\{\begin{array}{ll} \text{NA}, & \text{if}\;\text{WD}\leq R\;\text{tan}\;\theta \\ \frac{R\;\text{tan}\;\theta }{\text{WD}\;}\text{NA}, & \text{otherwise}\; \end{array}$$

where *n* sin *θ* = NA, *R* is the fibre radius, and *n* is the RI of the immersion medium. As an example, for a Thorlabs FP200ERT fibre (200 μm core, NA=0.50) and a RI=1.5, this yields a cylindrical volume of ∅100 μm and 140 μm height, if the NA of 0.5 is required across the whole imaged volume. Tolerating a factor of 2 in NA non-uniformity (NA within [0.25,0.5] range) yields a cylinder of ∅200 μm and 565 μm height, providing volumes for which optical clearing can be fully appreciated. While having significantly higher NA, and larger FOV and working distance, the number of modes at 532 nm in this fibre is about 130000, in contrast to about 1600 modes for the fibre used in this work. Imaging with such fibres would benefit from significantly faster SLMs and DMD or require longer calibration and acquisition times. Alternatively, direct wavefront measurements and digital phase conjugation approach [12] can be a suitable choice.

While we acknowledge that the present cost of the system proposed here is comparable to that of a specialized clearing-compatible objective lens (>$15k for the liquid crystal SLM), we note that the system offers an inherent flexibility in operating parameters compared to a fixed objective. The SLM may readily be replaced with a low-cost (∼$200–500) and fast (∼ 22 kHz) digital micro-mirror device (DMD) [26] if cost is an issue.

On the other hand, for single-photon fluorescence and spontaneous Raman scattering, wavefront shaping is not strictly necessary, as imaging can be performed with any excitation basis, of which focused spots is just one option. For example, scanning the beam angle on the fibre input produces pseudo-random illumination patterns at the sample plane, which can equally be used to reconstruct the image from a single-pixel ‘bucket’ detector, and even provide a two-fold gain in resolution similar to structured illumination microscopy [27]. Additionally, such random, nearly orthogonal bases are particularly suitable for compressive sensing applications [28], and thus can greatly reduce the number of required measurements. Here the scanning may be implemented simply with galvanometer mirrors, thus reducing the barrier to entry for many microscopy labs.

Our approach is readily generalized to graded index (GRIN) lenses, which are essentially precision-cut pieces of a GRIN fibre. They are widely available with higher NA than MMFs (0.4–0.6) and typically have larger diameter (>350 μm) and FOV. In contrast to fibres, they are designed to transmit undistorted images for given immersion RI (usually air, *n* = 1) and distance to the sample. The number of modes supported by GRIN lenses is orders of magnitude higher than that of the fibre used in this work, so direct acquisition of the full transmission matrix [11,29] is more challenging. However, due to their image transmission properties they only exhibit low order aberrations, and access to a smaller number of degrees of freedom is sufficient to provide suitable correction, which will only vary slowly across the FOV [30]. In the context of a complex medium, the image transfer property of GRIN lenses can be seen as a strong optical memory effect [31], which means that once the wavefront correction is performed to achieve focusing at one point, the whole FOV can be obtained by a simple beam scanning at the input. Therefore, a fixed phase mask placed before the complex medium can replace dynamic SLMs for given imaging conditions (RI, NA), significantly reducing the cost of the imaging system.

The advantage of optical clearing for imaging large volumes is mostly appreciated when using light-sheet microscopy (or single plane illumination microscopy, SPIM) [32], as it provides a dramatic increase in acquisition speed compared to scanning techniques. As shown recently in [18], MMF waveguides are compatible with the light-sheet modality, and an all-fibre endoscope operating similar to swept confocally-aligned planar excitation (SCAPE) principle [33] may also be envisaged.

To the best of our knowledge, we have shown the first demonstration of MMF imaging applied to optical clearing microscopy. More generally, our approach demonstrates that a lens of a monolithic design, coupled with wavefront correction of ‘complex media’, can extend the application range of an imaging system, bringing down the cost and complexity. Our work shows how a simple MMF can fulfil this role and circumvent the main challenges associated with the design of optical clearing objectives. The MMF probe is inexpensive, shows complete insensitivity towards immersion refractive index change, and has superior chemical stability. This paves the way for this technology to advance imaging in standard one- and two-photon microscopy, Raman, and light-sheet imaging for optically cleared samples.

## Appendix

### A1. Raman image analysis

As the sample spectra were contaminated by both Raman background of the MMF, and by the contribution of TDE, reference spectra were required. An internal reference was provided within the image scan itself, by the points not occupied by the sample compound (polystyrene in polystyrene beads), allowing for subtraction of background. However, the background-corrected Raman signal from polystyrene was intrinsically mixed with the TDE immersion contribution, as whenever a volume of polystyrene is present in the excitation cone of light, an equal volume of TDE is missing. Therefore, to extract the contribution of TDE Raman signal from the sample spectrum, an additional reference acquisition were performed, by scanning the focus across the glass-TDE interface.

All spectra for a given image formed a measurement matrix *A*, which was decomposed by principal component analysis (PCA) into coefficients (spectral components, *C*), and scores (amount of each spectral component present in a given image pixel, *S*): *A* = *C* · *S*. PCA reduces the effective dimension of the dataset, so only the few first components suffice to describe most of the variation in the data. However, as the principal components are discriminated and sorted merely based on their variance, they usually fail to identify the true Raman spectra, providing a linear combination of them instead. Therefore, we linearly transformed the basis corresponding to the first few components in order to restore the pure spectra. For a basis transform matrix *T*, a new set of coefficients and scores can be obtained, such that the resulting decomposition remains valid:

(2) $$C^{*}=C\cdot T,\;S^{*}=T^{-1}\cdot S,\;A=C\cdot S=C^{*}\cdot S^{*}$$

The transformation matrix was generated in order to obtain TDE and polystyrene spectra that would be both non-negative (Raman spectra cannot be negative, unlike principal components) and sparse (Raman spectra are mostly zero for TDE and polystyrene, in the 1100–2800 cm^−1^ region). The pixel intensities of the Raman images are thus the values of the vector *S*^*^. We emphasize that our method is different from the conventional Raman imaging, where a particular narrow spectral range (typically, a single vibrational mode) is used to construct the image.

### A2. Fluorescence imaging

#### A2.1. PMT with digital acquisition card

The output of the PMT (Hamamatsu H6780-20, 10^6^ gain, 1MΩ load) was recorded using a DAQ module (NI USB-6008). PMT signal was recorded as a continuous waveform, and an external synchronization signal was used to assign parts of the waveform to different pixels of the final image. As our SLM cannot provide a dedicated mask update sync signal, a region of the SLM which did not couple into the objective was used to generate such a signal for data post-processing. This SLM region was kept blank on even frames, while on the odd frames a blazed phase grating was used to divert the beam to an auxiliary photodiode (PD1), generating a square wave sync signal. Additionally, to account for the power fluctuations due to the laser and the SLM, a second photodiode (PD2) was used to monitor the instantaneous power going into the fibre.

#### A2.2. Spectrometer

Fluorescence spectra from stained cells were acquired at each image pixel, with the integration time of 0.1 s, and other settings (grating, slit width) identical to those of Raman acquisition. The collection of the spectra was treated by PCA, and the principal component corresponding to the best contrast of the cells was used to obtain the image.
